# Bacterial Lux Biosensors in Genotoxicological Studies

**DOI:** 10.3390/bios13050511

**Published:** 2023-04-29

**Authors:** Serikbai K. Abilev, Elena V. Igonina, Darya A. Sviridova, Svetlana V. Smirnova

**Affiliations:** The Laboratory of Ecological Genetics, Vavilov Institute of General Genetics, Russian Academy of Sciences, Moscow 117971, Russia; iev555@ya.ru (E.V.I.); daria_sv11@mail.ru (D.A.S.); s.v.smirnova.genet@gmail.com (S.V.S.)

**Keywords:** lux biosensors, *E. coli*, drugs, chemical agents, environmental pollutants, genotoxicity, antioxidants, deuterium

## Abstract

The aim of this study was to assess the applicability of the bacterial lux biosensors for genotoxicological studies. Biosensors are the strains of *E. coli* MG1655 carrying a recombinant plasmid with the *lux* operon of the luminescent bacterium *P. luminescens* fused with the promoters of inducible genes: *rec*A, *col*D, *alk*A, *sox*S, and *kat*G. The genotoxicity of forty-seven chemical compounds was tested on a set of three biosensors pSoxS-lux, pKatG-lux and pColD-lux, which allowed us to estimate the oxidative and DNA-damaging activity of the analyzed drugs. The comparison of the results with the data on the mutagenic activity of these drugs from the Ames test showed a complete coincidence of the results for the 42 substances. First, using lux biosensors, we have described the enhancing effect of the heavy non-radioactive isotope of hydrogen deuterium (D_2_O) on the genotoxicity of chemical compounds as possible mechanisms of this effect. The study of the modifying effect of 29 antioxidants and radioprotectors on the genotoxic effects of chemical agents showed the applicability of a pair of biosensors pSoxS-lux and pKatG-lux for the primary assessment of the potential antioxidant and radioprotective activity of chemical compounds. Thus, the results obtained showed that lux biosensors can be successfully used to identify potential genotoxicants, radioprotectors, antioxidants, and comutagens among chemical compounds, as well as to study the probable mechanism of genotoxic action of test substance.

## 1. Introduction

Biosensors are the organisms and a device that respond to environmental factors, and such responses could be as a specific biological activity or can be used to measure the concentration of various types of analytes. Luminescent bacteria, which are used to assess the integral toxicity of the environmental factors, are most widely used as biosensors [1]. To date, more than 1000 different tests based on the evaluation of the bioluminescence of various luminous microorganisms have been developed. The “Microtox” system, developed by Microbics Operations of Beckman Instruments Inc (USA) in the 1970s and 1980s, is used the most commonly [2]. Modified versions of this test system used for determining acute and chronic toxicity of chemicals are produced by AZUR Environmental (USA). They contain the marine bacterium *Vibrio fischeri* NRRL serving as a biosensor, which exhibits high sensitivity to a wide range of chemical pollution of industrial, waste and natural waters, and soil and bottom sediments [3]. There are other commercially available test systems, that are cost effective and easy to use to evaluate the levels of the environmental pollution, even in the field. However, these systems also have a number of disadvantages. To name just a few—the temperature limitations of the analysis (the measurement should be performed at low temperature (15 °C)), the presence of an osmoprotectant of marine bacteria, and the lack of specificity.

In order to overcome these limitations, the recombinant biosensors were developed with *E. coli* strains with a multicopy pPHL7 plasmid carrying the *Photobacterium leiognathi lux* operon [4]. Later, series of biosensors with complete operons *lux*CDABE of *Vibrio fischery*, *P. leiognathi*, and *Photorhabdus luminescens* in vector plasmids were created [5,6,7,8]. These recombinant *E. coli* strains are used in Russian “Ecolum” test system to determine the integral toxicity of environmental objects. This test system is supported by the certificate of the Committee for Standardization and Metrology of the Russian Federation No. 01.19.231/2000 [3]. The advantage of recombinant biosensors is the exclusion of osmotic correction of the studied samples, which makes them suitable for the analysis of fresh water and aqueous solutions of chemical compounds. Additionally, they do not have the abovementioned temperature limitations and could be used at temperatures 22–37 °C.

To enhance the specificity of the biosensor, a group of authors led by G.B. Zavilgelsky developed biosensors based on an *E. coli* strain carrying a plasmid with the *lux*CDABE operon placed under the control of inducible promotors [9,10,11,12]. The genes of *lux*CDABE operon are the most universal part of the whole jointly transcribed *lux* operon of all known luminescent bacteria [3]. *lux*AB determines the synthesis of the luciferase subunits, and *lux*CDE mediates the synthesis of the reductase subunits that participate in the generation of the luceferase substrate—tridecanal—as a result of the reduction of fatty acids [4].

To detect the agents capable of inducing oxidative stress in a cell, biosensors with a multicopy recombinant plasmid were developed, fusing the *lux* operon from *P. luminescens* with the promoters of the catalase and superoxide dismutase genes [9,12]. The protein OxyR specifically reacts to an increase in the concentration of hydrogen peroxide and other peroxides in the bacterial cell and activates the promoter of the *kat*G gene [6,13]. The activator protein SoxR of the *sox*S gene promoter specifically responds to the superoxide anion radical. In addition, lux biosensors for thermal shock detection have been proposed [5]. They contain *lux* operon under the inducible promoters of the *grp*E and *ibp*A genes. The expression is induced by the elevated temperature and, to a lesser extent, substances: ethanol, phenol, hydrogen peroxide, sodium azide, and others [9,10]. 

To study the genotoxicity of the environmental chemical factors, bacterial lux biosensors designed according to the principles described above are used. They are carrying plasmids with the *lux* operon under the control of the promoters of *rec*A and *cda* (*col*D) genes. In this case, genotoxicity is detected as a result of activation of the SOS response system in *E. coli* [9,10]. The SOS response, discovered in the bacterium *E.coli*, is a coordinated induction of approximately 40 genes, which occurs as a result of DNA damage by UV radiation, mitomycin C, bleomycin, etc. [14]. Important factors of the SOS response induction are single-strand DNA breaks occurring as a result of the blockade of DNA synthesis by DNA-damaging agents. The molecular mechanisms of the SOS response induction in *E. coli* cells have been studied in detail and reviewed elsewhere [14,15,16]. Thus, pRecA-lux and pColD-lux biosensors represent the expression of the starting and terminal genes of the SOS regulon in response to DNA damage by genotoxicants [16].

In 1982, a bacterial SOS chromotest was proposed to determine the DNA-damaging activity of chemical compounds [17]. It is a colorimetric assay of the SOS response based on the *E. coli* strain PQ37, where the *lac*Z gene is fused with the *sfi*A promoter that belongs to the SOS response system. The results of testing 741 chemical compounds using the SOS chromotest are given in the review [18]. An amount of 404 (54%) of these chemicals present a genotoxic activity detectable in the SOS chromotest. 

For the detection of metal and metalloid ions, as well as alkylating agents, highly sensitive specific lux biosensors based on *E. coli* were created. Bacteria contain hybrid plasmids pArsR’::lux, pMerR’::lux, pCopA’::lux, pAlkA’::lux, where the transcription of *P. luminescens lux*CDABE reporter genes is carried out from the inducible promoters: the *ars* operon (induction by arsenic and antimony ions), the *mer* operon (induction with mercury and cadmium ions), the *cop*A gene (induction with silver and copper ions), and the *alk*A gene (induction with alkylating substances), respectively [10].

For the detection of antibiotics in food, lux biosensors that specifically respond to the antibiotics of the tetracycline series have been proposed—*E. coli* MG1655Z1 (pTetA’::lux), β-lactam series—*E. coli* MG1655 ampC::kanr (pAmpC’::lux), on antibiotics of the quinoline group—*E. coli* MG1655 (pColD’::lux) and aminoglycosides—*E. coli* MG1655 (pIbpA’::lux). High specificity of these strains for the indicated groups of antibiotics was shown [11].

Lux biosensors based on the recombinant strains are used to detect the contamination of natural waters [19,20,21,22] and soil [23] with genotoxicants, heavy metals, oxidants, and other compounds. The use of lux biosensors for large-scale monitoring saves time for analysis, reduces material costs, and requires minimal equipment for reading luminescence, which undoubtedly makes this method in demand when it is necessary to evaluate a large number of samples.

It should be noted that recombinant whole-cell biosensors allow us to study the parameters of linear and nonlinear dependence of luminescence not only on the dose and duration of exposure to the inducer, but also on the metabolic state of the cell itself. Mathematical methods have been developed for analyzing and constructing models of biosensor luminescence in various cases specified by the experimenter during a 48-h reaction. For this purpose, the results of Cd luminescence of a sensitive biosensor were used. Based on the theory of the time reaction of metal-sensitive luminescent bacteria, the authors carried out successful theoretical reconstructions of bioluminescence signals at all studied Cd concentrations (0–20 nM) and various nutrition conditions [24,25].

Thus, various natural and recombinant luminescent bacteria have become a tool for environmental monitoring. Specific “reporter systems” that have expanded the scope of the luminescent analysis have been developed. They are based on the investigation of signaling pathways in bacteria. The identification and sequencing of the gene regulators of proteins included in different signaling systems made it possible to create recombinant luminescent bacterial biosensors [9,10,11].

This paper presents the main results of evaluation of applicability of the lux biosensors based on the *E. coli* MG1655 strain for studying the genotoxicity of a wide range of chemical compounds and investigation of the mechanisms of their genotoxic effects, antioxidant and radioprotective activities, as well as other factors modifying genotoxic activity.

## 2. Materials and Methods

### 2.1. Bacterial Strains

The strains were provided by Drs. Zavilgelsky and Manukhov, State Research Institute of Genetics and Selection of Industrial Microorganisms, Moscow (Table 1). *E. coli* MG1655 strains carrying a plasmid with promoters of inducible genes: *rec*A, *col*D, *alk*A, *sox*S, and *kat*G, transcriptionally linked to the *lux* operon *lux*CDABE of the luminescent bacterium *P. luminescens*. Strain genotypes and structures of the recombinant plasmids are reported in [9,10].

### 2.2. Test of Chemical Compounds for Genotoxicity

*Chemicals*. Mitomycin C, paraquat, cisplatin (cis-diamminedichloridoplatinum(II)), 4-nitroquinoline-1-oxide (4-NQO), N-nitroso-N-methylurea (NMU), metylmetansufonat (MMS) streptozotocin, 2-nitrofluorene, 9-aminoacridine, actinomycin D, ethidium bromide, 5-fluorouracil, 2-aminopurine, and 5-bromouracil were purchased from Sigma Chemical Co (USA). All metal compounds were purchased from Reakhim (Russia). Pharmaceuticals: Cifran (Ranbaxy, India), Fluimucil (Zambon, Italy), Duspatalin (Abbott Healthcare Products B.V., Netherlands), De-Nol (Astellas, Netherlands), Furamag and Furagin (Olainfarm, Latvia), Lipoic acid (Artesan Pharma GmbH and Co. KG, Germany), and reduced glutathione (AppliChem GmbH, Germany). Pharmaceuticals from Russian companies: Dioxidine (Moskhimfarmpreparaty), Metronidazole (Obnovlenie), Fluconazole (Verteks), Mexidol (Farmsoft), Furacilin (Tatkhimfarmpreparaty), Furazolidone (Marbiofarm), Azithromycin (Proizvodstvo medikamentov), Ceftriaxone (Deko), and Omeprazole (Ozon). Hydrogen peroxide (H_2_O_2_) were purchased from Pharmaceutical company “Ferein” (Russia). Dimethyl sulfoxide pure (DMSO, AppliChem). All the working solutions of the tested compounds were prepared immediately before use. All the chemical substances’ purity was analytical grade.

*Assessment of the SOS Response and Oxidative Stress*. The bacteria were grown in an LB broth with 100 μg/mL ampicillin. The overnight culture was diluted with fresh broth to 10^7^ cells/mL and grown at 37 °C for 2–3 h. Portions of 180 μL were loaded into wells of a 96-well plate (Perkin Elmer OptiPlate-96), then 20 μL of genotoxicant solution was added to the same wells (except for the control wells) and incubated for 90 min. The substances were tested in 5 concentrations. The control wells contained the corresponding solvent: water or DMSO solution. Luminescence after 90 min of incubation was measured using a Beckman coulter DTX 880 microplate reader ((Beckman Coulter Inc., Fullerton, CA, USA) at room temperature. The readings were expressed in relative light units (RLUs). In the preliminary experiments, the toxic concentrations of the compounds tested were assessed from the suppression of the cultures’ luminescence. The principal parameter of luminescence induction was taken to be the induction factor IF (induction factor), determined as: IF = I_ind_/I_0_, where I_0_ is the spontaneous luminescence of the culture without an inducer, and I_ind_ is luminescence with the presence of the substance tested. A statistically significant excess of I_ind_ over I_0_ was assessed using Student’s test and considered as the validity criterion *p* ≤ 0.05. All the experiments were carried out in four parallel wells. Each substance was tested in three independent replications.

### 2.3. The Modifying Effect of Deuterium on the Activity of Chemical Genotoxicants 

*Chemicals.* Deuterium oxide (D_2_O) was produced by Chemical Line (Russia). Mitomycin C, 4-NQO, and NMU (from Serva, Germany). Sources of other chemicals and drugs used are given in Section 2.2.

*Assessment of effect of D_2_O on the activity of chemical genotoxicants*. An overnight *E. coli* culture was grown in a complete LB medium containing ampicillin (100.0 μg/mL). On the day of the experiment, the overnight culture was diluted with a fresh medium to a density of (1–2) × 10^6^ cells/mL. The measurements were made with a DEN-1B densitometer (BioSan, Latvia). Then, the cells in the suspension were let to grow to the early logarithmic phase (37 °C, 2 h, on a shaker at 120 rpm). Aliquots of this culture (160 μL) were transferred into sterile wells of a 96-well plate and supplemented with 20 μL D_2_O-containing water the final concentration of D_2_O ranged from 5 to 10% in different wells. Then, the plates were incubated for 90 min at 37 °C for pre-deuteration of bacteria. The control wells were supplemented with distilled “light” water H_2_O. Then, 20 μL of genotoxicant solution was added to the same wells (except for the control wells) and incubated for 90 min. The luminescence after 90 min of incubation was measured using a Stat Fax 4400 microplate reader (Awareness Technology, USA). Experiments were performed at least three times in eight replicates. The results were expressed in relative light units (RLU). Statistical treatment of the data was carried out in the Data Analysis Tool Pack add-in in Microsoft Excel, StatPlus, and WINPIPI. Statistical significance of the mean differences was assessed using Student’s test. The deuterium isotope effect (IE) was evaluated as the ratio of the luminescence of the biosensors with deuterium (I_D_) to that of the biosensors without deuterium (I_0_).

### 2.4. A Study of the Pro- and Antioxidant Activity of Substances from Different Sources 

*Antioxidants:* reduced glutathione and biotin (AppliChemGmbH, Germany); cysteine, acetylcysteine, methionine, cystine, and DMSO (Serva, Germany); dihydroquercetin (abcrGmbH, Germany); taufon (taurine) (Moscow Endocrine Plant, Russia); lipoic (thioctic) acid (Worwag Pharma, Germany); ascorbic acid (Meligen, Russia); spermine (Acros Organics, United States); mexidol (Farmasoft, Russia); nicotinic acid (Obnovlenie, Russia); pyridoxine (CSPC Ouyi Pharmaceutical Co. Ltd., Shijiazhuang, China); lycopene and coenzyme (Evalar, Russia); vitamin A (Lumi, Russia); and vitamin E (Farmabiofarm, Russia). 

*Radioprotective agents:* B-190 (indralin), synthetic genistein, glutathione disulfide, magnesium salt, lithium salt of glutathione disulfide and zinc salt of reduced glutathione (Farmzaschita, Russia); glutoxim (glutathione disulfide disodium salt with platinum at nanoconcentrations) and molixan (complex of glutoxim with nucleoside inosine) (Farmavit, Russia); cystamine (cystamine dihydrochloride) (Farmakon, Russia); recrystallized cysteamine (State Research Institute of Military Medicine) and 5-androstenediol substance (5-AED, Hollis-Eden Pharmaceuticals, San Diego, CA, USA); H_2_O_2_ (Pharmaceutical company Ferein, Russia) and paraquat (Serva, Germany) were used as oxidative stress inducers. All test solutions were prepared immediately before use. 

*Measurement of the luminescent reaction of lux biosensors.* Overnight cultures of pKatG-lux and pSoxS-lux biosensors were diluted to 10^7^ cells/mL in fresh LB medium and grown with aeration at 37 °C to the early exponential phase. Then, 160-μL aliquots of the sample were transferred to the wells of a 96-well plate. The control wells were supplemented with 40 μL of distilled water, and the experimental wells were supplemented with 20 μL of the antioxidant solution at various concentrations and 20 μL of H_2_O_2_ (in the case of the pKatG-lux sensor) or paraquat solution (in the case of the pSoxS-lux sensor) to the final concentrations of 0.001 and 0.0004 mmol/L, respectively. Then, the plates were incubated for 45 min (in the case of pKatG-lux) or 60 min (in the case of pSoxS-lux). Experiments were performed at least three times in eight replicates. 

The luminescence intensity was measured in a StatFax 4400 microplate reader (Awareness Technology Inc., Palm City, FL, USA). The bioluminescence intensity was expressed in relative light units (RLU). The effect of the analyzed substances on the peroxide- or paraquat-induced luminescence of the sensors was evaluated using the following formula:

$$AA=\left(1-\frac{I_{a}}{I_{p}}\right)\times 100$$

where *AA* is the protective (antioxidant) activity, *I_p_* is the biosensor luminescence intensity induced by hydrogen peroxide or paraquat, and *I_a_* is the biosensor luminescence intensity induced by hydrogen peroxide or paraquat in the presence of an antioxidant. This formula reveals the antioxidant (protective) or, conversely, prooxidant stimulating activity of the substance under investigation.

## 3. Results

### 3.1. Genotoxicity Analysis of Chemical Compounds Using a Set of Three Biosensors: pSoxS-lux, pKatG-lux, and ColD-lux

Forty-seven chemical compounds were tested for genotoxicity by a set of biosensors: pSoxS-lux, pKatG-lux, and pColD-lux, carrying a recombinant plasmid with the *lux* operon of the luminescent bacterium *P. luminescens*, fused with promoters of superoxide dismutase *sox*S, catalase *kat*G, and colicin *col*D genes. This set of biosensors makes it possible to evaluate the oxidative and DNA-damaging activities of the studied drugs. According to the chemical structure and field of application, the tested compounds could be conditionally classified into several groups: metal salts, containing a nitro group, base analogs intercalating in DNA, antitumor, antibacterial, and various drugs.

Hydrogen peroxide, paraquat, mitomycin C, and 4-NQO were used as reference genotoxicants. Hydrogen peroxide and paraquat induce oxidative stress in pKatG-lux and pSoxS-lux biosensors. Mitomycin C and 4-NQO cause crosslinking and adducts in DNA of pCol-lux, respectively [26,27].

The substances were tested in five concentrations (Table 2). The test result of the substances is presented as IF—ratio of the induced luminescence to the relevant control in concentrations of maximum effect. The concentrations of substances at which the indicated IF are obtained are given in parentheses. The results of studying the activity of chemical compounds in the mutation test are given in the “Ames test”.

Nitro compounds, except for the 2-nitrofluorene, exhibited high genotoxicity and induced the SOS response in the pColD-lux biosensor. Of the 10 metal salts evaluated for their toxicity, only CdCl_2_ and K_2_Cr_2_O_7_ increased the concentration of superoxide and peroxide in the pSoxS-lux and pKatG-lux biosensor cells, respectively. Bactericidal agents dioxidine and ciprofloxacin activated the pCold-lux biosensor. The base analogs 2-aminopurine and 5-bromuracil, the alkylating agents NMU, streptozotocin, and cisplatin also activated the pColD-lux biosensor. Intercalating agents 9-aminoacridine, ethidium bromide and acridine orange did not activate the pColD-lux biosensor. Among the various substances, only duspatalin and iodine showed the activation of the pKatG-lux and pSoxS-lux biosensors, respectively. Twenty substances showed activation of the tested biosensor set: sixteen substances induced the SOS response in ColD-lux and five substances induced oxidative stress: two—in pKatG-lux, one—in pSoxS-lux, two—in pKatG-lux and pSoxS-lux.

### 3.2. Modifying Effect of Deuterium on the Activity of Chemical Genotoxicants and Mutagens

Deuterium is a stable isotope of hydrogen with similar physical and chemical characteristics. The deuteration reaction is the replacement of covalently bonded hydrogen atom with a deuterium atom [50]. Thus, selective deuteration of a drug keeps the pharmacologic effect [51]. Pharmaceutical companies investigate deuterated agents as new chemical entities to bring favorable pharmacokinetic properties. The replacement of hydrogen with deuterium effectively increases the drug’s metabolic stability by prolonging the half-life, allowing for reducing the dose, which provides better safety and efficacy [51,52].

In this regard, we decided to test the ability of deuterium to influence the inducible processes in bacterial cells. First, we used the reference mutagens 4-NQO, NMU, and mitomycin C capable of inducing the SOS response in bacteria bearing pColD-lux biosensor (Table 2). To study the effect of bacterial pre-deuteration on the genotoxicity of chemical compounds, we used deuterium oxide (D_2_O) and pRecA-lux and pColD-lux biosensors. 

**Figure 1:**
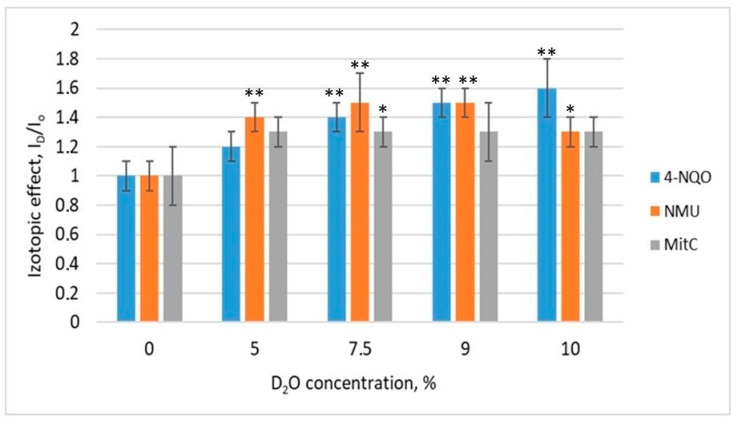
Isotopic effect of D_2_O on the induction of the SOS response in pRecA-lux biosensors exposed to 4-NQO (8 × 10^−4^ M), NMU (2 × 10^−2^ M), and Mitomycin C (5 × 10^−6^ M). Asterisks correspond to comparisons with control (zero concentration D_2_O): *—*p* < 0.05, **—*p* < 0.01.

**Figure 2:**
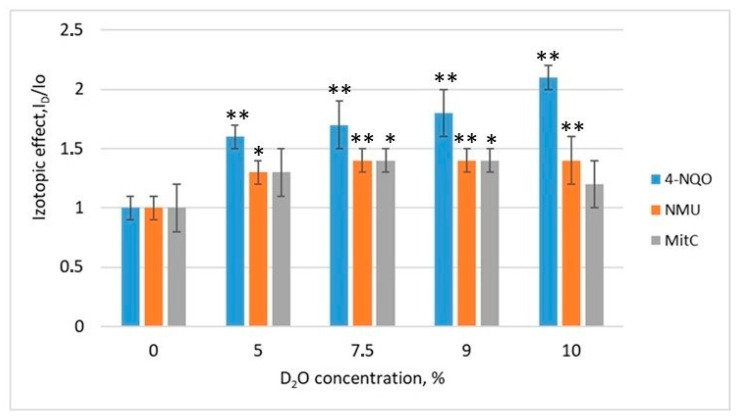
Isotopic effect of D_2_O on the induction of the SOS response in pColD-lux biosensor exposed to 4-NQO (8 × 10^−4^ M), NMU (2 × 10^−2^ M), and mitomycin C (5 × 10^−6^ M). Asterisks correspond to comparisons with control (zero concentration D_2_O): *—*p* < 0.05, **—*p* < 0.01.

The isotopic effect of D_2_O on the induction of the SOS response in pRecA-lux and pColD-lux biosensors using 4-NQO, NMU, and mitomycin C is shown in (Figure 1 and Figure 2).

Mitomycin C, 4-NQO, and NMU induce SOS response in biosensor cells, differing in the mechanisms of DNA damage. Namely, after the reduction by bacterial nitroreductase, 4-NQO forms an adduct with DNA [53], NMU alkylates DNA [54] and mitomycin C crosslinks into DNA chains [55].

For all the genotoxicants used, an increase in the luminescence levels of the pRecA-lux and pColD-lux biosensors was observed when they were pre-deuterated. The isotopic effect is the ratio of the luminescence of the biosensors stimulated with deuterium (I_D_) to that of the biosensors without deuterium (I_0_). The addition of D_2_O in absence of genotoxicants did not cause any response of the test systems. It is clear that the magnitude of the deuterium isotope effect depended on the type of genotoxicant as well as on the inducible promoter used at biosensor. The maximum values of the I_D_/I_0_ coefficient were observed at the D_2_O concentration of 7.5% (I_7.5_/I_0_), whereas the decrease in biosensor luminescence was observed at a D_2_O concentration of 10%. It was probably due to the effect of D_2_O on the activity of proteins of the luminescence system or to the synergy of the toxic effects of D_2_O and genotoxicants.

Thus, for the first time we obtained the data on the reinforcing effect of deuterium on the induction of SOS response in biosensors by the reference compounds. Therefore, to study the effect of deuterium on the genotoxicity of substances other than the reference mutagens, we have analyzed base analogues (2-aminopurine, 5-bromuracil), a helicase inhibitor nalidixic acid, nitroheterocyclic compounds 4-NQO and furacilin (nitrofural), oxidative stress enhancer dioxidin, as well as antitumor agents cisplatin and streptozotocin (Table 3).

### 3.3. The Effect of Deuterium on the Adaptive Response of E. coli to Genotoxic Effects of the Alkylating Agents

It is well known that both prokaryotes and eukaryotes utilize a system of protection against toxic and genotoxic effects of alkylating agents called adaptive response (AR). Specifically, pre-exposure of bacteria to nontoxic levels of alkylating agents leads to the increase in their tolerance to higher levels of these agents [56,57,58]. AR is a direct repair of alkylated DNA bases. In *E. coli*, the AR system includes the *ada*, *alk*A, *alk*B, and *aid* genes combined into the *ada* regulon, and their expression is controlled by the Ada protein [55].

We have evaluated the effect of D_2_O on the *alk*A gene expression induced by the alkylating mutagens NMU and MMS using pAlkA-lux biosensor. Analysis of the results obtained showed that pre-deuteration of the AlkA-lux for 90 min led to an increase in the AR level after induction with NMU and MMS in concentrations 0.005 mol/L (Table 4). The isotope effect depended both on the alkylating agent and on the D_2_O concentration. The highest isotope effect was observed at the D_2_O concentrations of 7.5 and 9% (Table 4). At the D_2_O concentration of 10%, a decrease in the biosensor luminescence was observed. The observed effect could be associated with the influence of D_2_O on the activity of the luminescence system proteins or with the synergistic toxic effect of D_2_O and alkylating compounds. This is the subject of further research. 

In the biosensor culture deuterated with 7.5% D_2_O, the activity of MMS and MNU at a concentration of 0.005 mol/L increased more than five- and three-fold compared to the activity on a non-deuterated culture. 

### 3.4. Testing for Pro- and Antioxidant Activity of Substances from Various Sources by pSoxS-lux and pKatG-lux Biosensors 

We have evaluated the effects of 29 substances, including known antioxidants, anti-radiation agents, amino acids, and vitamins, on the oxidative stress in pSoxS-lux and pKatG-lux biosensors induced by paraquat and H_2_O_2_, respectively. The luminescence of these biosensors is the result of the activation of the *sox*S and *kat*A gene promoters in response to an increase in the concentration of superoxide radical and H_2_O_2_ in the cell. In the case of the antioxidant action of the studied substance, the intensity of induced luminescence in biosensors decreases, and in the case of a prooxidant action, it increases. 

In this experiment, H_2_O_2_ and paraquat were used at the final concentrations of 0.001 and 0.0004 mmol/L, respectively. Plates with the biosensors were incubated at 37C for 60 min. Antioxidant activity was shown by 23 of the 29 substances (79%) on the pKatG-lux biosensor and by 22 of the 29 substances (76%) on the pSoxS-lux biosensor. The studied anti-radiation agents (10 substances) showed different degrees of pro- and antioxidant activity (Table 5).

High prooxidant activity among the anti-radiation agents on the pKatG-lux biosensor at low concentrations was shown by lithium and magnesium salts of glutathione disulfide, zinc salt of reduced glutathione, molixan, and indralin (B-190); on the pSoxS biosensor—by genistein, cystamine, and Androstenediol (AED). 

Almost all vitamins exhibited both prooxidant and antioxidant activity, except for the vitamin C (ascorbic acid) and vitamin E, which exhibited high antioxidant activity in both biosensors (i.e., suppressed the oxidative stress in bacterial cells induced by hydrogen peroxide and paraquat). Vitamin C at concentrations of 10 and 20 mmol/L suppressed oxidative stress in pKatG-lux and pSoxS-lux cells by 99 and 95%, respectively. Vitamin B also suppressed the oxidative stress in both biosensor cell lines. Vitamin B at a concentration of 30 mmol/L suppressed oxidative stress in both biosensor cell lines. However, it showed prooxidant activity at concentrations of 12 and 5 mmol/L on biosensors in pKatG-lux and pSoxS-lux, respectively. 

Vitamin A at a concentration of 30 mmol/L was a prooxidant in the pKatG-lux biosensor, and at a concentration of 70 mmol/L, was an antioxidant in the pSoxS-lux biosensor. Vitamin PP at a concentration of 5 mmol/L showed antioxidant activity in the pKatG-lux biosensor. Biotin exhibited primarily prooxidant activity in both biosensors and a slight antioxidant activity in the pKatG-lux biosensor. 

Among the amino acids, cysteine at a concentration of 20 mmol/L showed a high antioxidant activity and suppressed oxidative stress by more than 90%. However, at low concentrations 1 and 5 mmol/L, cysteine showed prooxidant activity. Methionine, which also contains a sulfhydryl group in its structure, showed antioxidant activity at a concentration of 30 mmol/l only in the pSoxS-lux biosensor and weak prooxidant activity at a concentration of 1 mmol/l on the same biosensor. The activity of taurine was similar to the activity of methionine, except for its weak antioxidant activity which was detected in the pKatG-lux biosensor. Cystine and cysteine disulfide exhibited no antioxidant activity and behaved as a prooxidant in both biosensors.

Coenzyme Q10 at a concentration of 10 mmol/L showed antioxidant activity in both biosensors and was a weak prooxidant in the pKatG-lux biosensor. Mexidol, which protects cell membrane lipids from peroxidation, at a concentration of 36 mmol/L showed a high antioxidant activity in both pKatG-lux and on pSoxS-lux biosensors. However, at low concentrations, it showed the ability to generate the superoxide radical in the bacterial cell. Solvent DMSO, which is known in medicine as Dimexide, at a concentration of 100 mmol/L also showed antioxidant activity in both biosensors; however, at low concentrations (0.5 mmol/L), it showed prooxidant properties in the pSoxS-lux biosensor.

## 4. Discussion

From 2016 to 2022 we have conducted large-scale studies of genotoxicity of a wide range of organic and inorganic chemical compounds using bacterial lux biosensors. 

A comparison of the test results for the 47 chemical compounds with the results of evaluation of their mutagenic activity in the standard Ames *Salmonell*a/microsome bacterial test showed an agreement of the results for 42 substances. The Salmonella strains used in the analysis have different mutations in various genes in the histidine operon; each of these mutations was designed to respond to mutagens with different mechanisms of action. The principle of the reverse mutation test is that it detects the revert mutations of the strains to histidine independence. The Ames test is used worldwide as an initial screen to determine the mutagenic potential of new chemicals and drugs because there is a high predictive value for rodent carcinogenicity when a mutagenic response is obtained [28,37,59].

A comparison of the results of the 16 genotoxicants tested on the pColD-lux biosensor coincided with the data of the analysis of these substances in the SOS chromotest [25]. pColD-lux activation is recorded by the luminescence intensity, while the response of the *E. coli* PQ37 strain is recorded by the results of the biochemical reaction of galactosidase with the ONPG substrate (o-nitrophenyl-β-D-galactopyranoside) [26].

The lux test for the SOS response of *E. coli* is more convenient than the SOS chromotest in terms of technical performance and cost effectiveness, since the response is recorded by the glow of bacteria and does not require additional manipulations, such as the lysis of the bacteria after incubation or the determination of the enzymatic activity. 

The direct measurement of bacterium luminescence allows simultaneous recording of the dependence of the SOS response on the concentration of the compound tested and on the duration of incubation, i.e., the dynamics of the response. The test is highly sensitive and rapid. The results are available within 3–4 h. Moreover, in contrast to the Ames test, it can be automated.

The heavy hydrogen isotope deuterium (D) was discovered in 1932 [60], and the studies of its biological activity were started. The deuterium oxide (D_2_O) named “heavy water” has a negative effect on living organisms: it slows down metabolism; causes a decrease in the levels of protein and nucleic acid synthesis; inhibits mitosis at the prophase, which leads to disruption of the process of cell division and morphological changes; and decreases the rate of enzymatic reactions [48,58]. For instance, in experiments with tumor cells both in vitro and in vivo, D_2_O suppressed their division [61,62,63]. Despite numerous studies on the toxic effect of D_2_O at the level of the whole organism and on the influence on physiological and biochemical processes in the cell, there is insufficient information on its effect on genetic processes, such as mutagenesis and repair of damaged DNA. 

We have shown that D_2_O enhances the effects of genotoxicants on the activation of transcription from the promoters of the genes of DNA repair pathways. One of the simplest possible explanations for this phenomenon could be the enhanced strength of the genotoxicant–DNA bond in deuterated regions of interacting molecules. It is also possible that deuteration reduces the activity of the repair enzymes, leading to a decrease in the rate of repair of DNA regions damaged by the genotoxicant. Consequently, a disbalance arises between the rate of damage accumulation and the rate of repair of the initial DNA structure by the repair enzymes, leading to the accumulation of damage in the DNA structure. As a result, the SOS response in a cell is activated, providing the repair of the damaged DNA regions [64,65].

Deuterium can affect the metabolism of substances in the cell by changing the activity of certain enzymes. D_2_O increased the expression of the *rec*A gene and decreased the expression of the *ka*tG gene induced by peroxide in biosensors in pRecA and pKatG-lux biosensors. This means that the D_2_O background inactivation of the catalase by the H_2_O_2_ in the cell slows down and, consequently, the pool of hydroxyl radicals causing DNA single-strand breaks increases. The latter circumstance leads to the induction of the SOS DNA repair in a bacterial cell, which is registered by an increase in the expression of the *rec*A gene [66].

A simultaneous study of the luminescence intensity and survival rate of pCol-lux bacteria showed that the UV irradiation of D_2_O stimulated cells at a dose of 12 J/m^2^ enhances the luminescence intensity of viable biosensor cells by 2.5 times [67].

We have shown for the first time that deuterium enhances the repair of alkylated DNA bases (Table 4). This type of repair is called an adaptive response and differs in its mechanism from the SOS response, and it begins with the activation of the Ada protein as a transcription regulator. It was demonstrated that alkylation of the N-Ada domain increased its affinity for DNA from 100 to 1000 times [56,57,58]. This may play a considerable role in the D_2_O isotope effect. Replacement of protium by deuterium in these DNA sequences or in the protein itself may be one of the main factors in stabilizing the bond between the promoter and the alkylated protein and, hence, the enhancement of the Ada-regulon transcription. 

Thus, we found that D_2_O can modify the genetic effects of chemical mutagens, and this is due to a change in the activity of certain enzyme systems in deuterated cells involved in the biotransformation of the mutagen itself or the repair of DNA damage. In this regard, the heavy non-radioactive hydrogen isotope deuterium can be considered as a comutagen, which, without its own mutagenic activity, can enhance the mutagenic activity of other substances.

The fact that some substances, including radioprotective agents, exhibit prooxidant activity, is worth mentioning. This phenomenon is not an exception. It is well known that, in certain experimental conditions, antioxidants (thiols (including glutathione), ascorbic acid, quercetin, gossypol, miretsitin, etc.) exhibit prooxidant activity, which leads to the oxidative damage of cells [68,69,70,71]. Here, the redox status of the nutrient medium that is used for growing the bacterial cultures or cells plays an important role. 

In general, the lux biosensors we used showed high efficacy in the evaluation of the pro- and antioxidant activity of the 29 substances that belong to the different classes of chemical substances and are used for different purposes. 

Based on the results of the research, it was concluded that the test system of two biosensors (pSoxS-lux and pKatG-lux) is applicable for the initial assessment of the potential antioxidant and radioprotective activity of chemical compounds.

In mammals, approximately 20 redox-sensitive systems were described, which respond to the alterations of the ratio of reduced and oxidized SH-groups in proteins, leading to oxidative stress. Among such systems, the Nrf2 transcription factor occupies a special place. This factor activates the gene expression through the interaction with the cis-regulatory element ARE (antioxidant responsive element) [72,73,74]. 

At the same time, a bacterial cell cannot replace a mammalian cell, because it does not have the Nrf2/ARE system. However, the bacterial system is characterized by a high efficiency, does not require special reagents for biochemical reactions, and could be used successfully as a test system in screening the large arrays of chemicals for primary selection of potential antioxidants and radioprotectors.

## 5. Conclusions

A comparison of the test results, obtained for the 47 chemical compounds, with the results of their mutagenic activity in the standard Ames *Salmonella/*microsome bacterial test showed an agreement of the results for the 42 substances. For the first time, using lux biosensors, the influence of the heavy non-radioactive hydrogen isotope D_2_O on the genotoxicity of the chemical compounds was discovered. Analysis of the 29 antioxidants and radioprotectors using the pSoxS-lux and pKatG-lux lux biosensors showed that these biosensors could be successfully used for the initial assessment of the potential antioxidant and radioprotective activity of the chemical compounds. In general, the lux biosensors we used, based on *E. coli* strains and carrying a recombinant plasmid with the *lux* operon of the luminescent bacterium *P. luminescens* fused with promoters of various inducible genes, could be used to test a wide range of chemical compounds for the genotoxicity and the factors modifying it.

## Figures and Tables

**Table 1:** Bacterial Strains.

Strains *E. coli*	Biosensor Designation in the Article	Promotor of Gene	*Lux* Operon	Reference Compounds
MG1655(pKat::lux)	pKatG-lux	*kat*G	*lux*CDABE	H_2_O_2_
MG1655(pSoxS::lux	pSoxS-lux	*sox*S	*lux*CDABE	Paraquat
MG1655(pColD::lux)	pColD-lux	*col*D	*lux*CDABE	4-NQO
MG1655(pAlkA::lux)	pAlkA-lux	*alk*A	*lux*CDABE	MMS
MG1655(pRecA::lux)	pRecA-lux	*rec*A	*lux*CDABE	4-NQO

**Table 2:** Induction of luminescence in biosensors: pSoxS-lux, pKatG-lux, and pColD-lux by biologically active substances and heavy metal salts.

No.	Agent	Concentration, 10^−3^ M	IF, I_ind_/I_0_ *			Ames Test
			pCol-lux	pKat-lux	pSox-lux	
1	H_2_O_2_	0.01–2.2	10.01 (0.04) 3.7 × 10^−9^ **	18.53 (0.02) 2.2 × 10^−7^	8.60 (0.04) 6.5 × 10–8	+ [28]
2	Paraquat	0.04–2.0	– ***	2.32 (2.0) 2.5 × 10^−9^	4.39 (1.8) 1.0 × 10–7	+ [28]
3	Mitomycin C	0.001–0.1	13.80 (0.1) 8.8 × 10^−5^	–	–	+ [28]
4	4-NQO	0.05–1.3	41.80 (0.3) 3.7 × 10^−5^	15.88 (0.5) 6.5 × 10^−9^	4.29 (0.5) 4.7 × 10–6	+ [28]
5	2-Nitrofluorene	0.02–0.7	–	–	–	+ [28]
6	Furacilin	0.50–5.0	2.27 (1.3) 1.2 × 10^−5^	2.46 (2.5) 2.9 × 10^−6^	–	+ [28]
7	Furamag	0.50–5.0	5.73 (0.2) 2.0 × 10^−5^	–	–	+ [28]
8	Furagin	0.50–5.0	5.52 (0.5) 8.4 × 10^−6^	–	–	+ [28]
9	Furazolidone	0.50–5.0	4.53 (2.2) 3.4 × 10^−5^	–	–	+ [28]
10	Metronidazole	0.1–5.3	3.18 (5.3) 4.3 × 10^−7^	–	–	+ [28]
11	Cadmium(II) chloride (CdCl_2_)	0.05–1.0	–	2.95 (0.1) 2.5 × 10^−6^	2.08 (0.1) 4.8 × 10–5	+ [29]
12	Cadmium(II) bromide (CdBr_2_)	0.01–0.1	–	–	–	- [30]
13	Caesium chloride, (CsCl)	0.05–1.0	–	–	–	- [31]
14	Manganese(II) chloride MnCl_2_)	0.05–1.0	–	–	–	- [31]
15	Zinc sulfate (ZnSO_4_)	0.05–1.0	–	–	–	- [31]
16	Copper(II) sulfate (CuSO_4_)	0.01–1.0	–	–	–	- [32]
17	Cobalt(II) sulfate (CoSO_4_)	0.03–0.6	–	–	–	- [32]
18	Iron(II) sulfate (FeSO_4_)	0.01–0.1	–	–	–	- [32]
19	Chromium potassium sulfate CrK(SO_4_)_2_	0.01–0.1	–	–	–	- [32]
20	Potassium dichromate(VI) (K_2_Cr_2_O_7_)	0.03–0.6	–	–	5.34 (0.6) 5.0 × 10–7	+ [32]
21	Dioxidine	0.45–4.50	11.47 (0.9) 8.9 × 10^−7^	–	–	+ [32]
22	Ciprofloxacin	0.04–41.0	2.70 (0.04) 2.7 × 10^−5^	–	–	+ [33]
23	Ceftriaxone	0.02–1.8	–	–	–	- [34]
24	Azithromycin	0.01–6.0	–	–	–	- [35]
25	Fluconasole	0.03–32.0	–	–	–	- [36]
26	2-Aminopurine	0.05–5.0	3.06 (5.0) 1.3 × 10^−8^	–	–	+ [37]
27	5-Fluorouracil	0.08–38.0	–	–	–	- [28]
28	5-Bromouracil	0.05–5.0	2.84 (5.0) 6.6 × 10^−6^	–	–	+ [37]
29	5-Fluorodeoxyuridine	0.02–0.4	–	–	–	- [38]
30	9-Aminoacridine	0.04–3.0	–	–	–	+ [28]
31	Ethidium bromide	0.03–1.2	–	–	–	+ [28]
32	Acridine Orange	0.04–37.0	–	–	–	+ [28]
33	Nitrosomethylurea	0.01–48.5	11.00 (48.5) 2.4 × 10^−7^	–	–	+ [28]
34	Streptozotocin	0.04–1.8	7.41 (0.4) 4.8 × 10^−6^	–	–	+ [28]
35	Cisplatin	0.02–1.6	4.43 (0.8) 7.7 × 10^−6^	–	–	+ [39]
36	Duspatalin	0.02–42.0	–	4.33 (0.8) 0.3 × 10^−5^	–	- [40]
37	De Nol (bismuth subcitrate potassium)	0.01–1.4	–	–	–	- [41]
38	Omeprazole	0.03–5.0	–	–	–	- [42]
39	Iodine	0.04–15.0	3.99 (0.04) 5.2 × 10^−7^	–	–	- [43]
40	Glutathione reduced	0.03–3.2	–	–	–	- [44]
41	Lipoic acid	0.001–0.06	–	–	–	- [45]
42	Mexidol	0.02–18.0	–	–	–	- [46]
43	Fluimucil	0.06–60.0	–	–	–	- [47]
44	Taufon	3.2–31.0	–	–	–	- [48]
45	1,3- dicyclohexyl carbodiimide	0.1–15.0	–	–	–	- [49]
46	Ethanol	16–162	–	–	–	- [23]
47	Dimethyl sulfoxide	1.4–14.0	–	–	–	- [23]

* The ratio of induced luminescence by substances to luminescence in the control. **—Significance level values *p* < 0.05. ***—No induction of luminescence.

**Table 3:** Isotopic effect of D_2_O on the SOS-response pColD-lux and pRecA-lux biosensors to chemical genotoxicants.

Chemicals, mol/L	pColD-lux				pRecA-lux			
	*Isotope Effect *, I_D_/I_0_				*Isotope Effect *, I_D_/I_0_			
	5%	7.5%	9%	10%	5%	7.5%	9%	10%
Mitomycin C, 5 × 10^−8^	1.61 ** 9.2 × 10^−6^	1.70 2.9 × 10^−7^	1.75 4.1 × 10^−6^	1,52 6.2 × 10^−5^	1.29 5.1 × 10^−8^	1.30 7.9 × 10^−8^	1.34 4.5 × 10^−4^	1.25 4.5 × 10^−4^
Dioxidine, 10^−5^	1.43 4.5 × 10^−5^	1.54 3.9 × 10^−7^	1.57 5.9 × 10^−6^	1.36 6.3 × 10^−5^	- ***	-	-	-
Furacilin, 2.5 × 10^−3^	2.0 8.2 × 10^−4^	4.0 1.8 × 10^−9^	2.8 3.1 × 10^−4^	2.7 5.3 × 10^−7^	2.2 9.4 × 10^−13^	2.5 3.8 × 10^−11^	1.4 1.0 × 10^−6^	0.9 2.7 × 10^−2^
4-NQO, 8 × 10^−5^	1.86 9.8 × 10^−7^	1.97 2.9 × 10^−6^	1.98 1.7 × 10^−5^	2.08 4.3 × 10^−9^	1.49 5.4 × 10^−6^	1.63 1.8 × 10^−4^	1.82 1.0 × 10^−9^	1.69 3.0 × 10^−8^
Nalidixic acid, 10^−3^	1.68 7.1 × 10^−5^	2.31 9.4 × 10^−12^	2.36 7.0 × 10^−7^	2.21 1.8 × 10^−9^	1.33 2.2 × 10^−8^	1.40 1.1 × 10^−9^	1.24 2.3 × 10^−3^	-
Cisplatin, 5 × 10^−5^	1.31 1.7 × 10^−4^	1.36 1.4 × 10^−5^	1.44 5.4 × 10^−6^	1.27 1.6 × 10^−4^	1.16 2.6 × 10^−5^	1.16 1.2 × 10^−4^	1.16 6.0 × 10^−5^	1.09 5.2 × 10^−3^
Streptozotocin, 10^−5^	1.16 6.6 × 10^−3^	1.24 1.9 × 10^−2^	1.35 2.2 × 10^−5^	1.51 2.0 × 10^−7^	1.11 3.1 × 10^−3^	1.13 2.5 × 10^−2^	1.16 1.2 × 10^−3^	1.14 1.0 × 10^−3^
2-aminopurine, 2.5 × 10^−3^	-	1.49 5.4 × 10^−5^	1.46 7.9 × 10^−5^	1.51 6.5 × 10^−5^	1.16 1.1 × 10^−2^	1.34 1.7 × 10^−6^	1.63 1.2 × 10^−5^	1.22 6.4 × 10^−4^
5-bromouracil, 10^−2^	-	-	1.10 4.6 × 10^−2^	1.23 8.0 × 10^−4^	-	0.82 3.7 × 10^−2^	0.77 1.0 × 10^−3^	0.75 6.5 × 10^−4^

* I_D_ is the luminescence level of the culture pre-deuterated with different concentrations of D_2_O in the medium (5%, 7.5%, 9%, 10%). I_0_ is the luminescence level of non-deuterated culture. **—Significance level values *p* < 0.05. ***—No deuterium effects.

**Table 4:** The effect of D_2_O on adaptive response pAlkA-lux biosensor induced by NMU and MMS.

Variant	D_2_O Concentration in the Medium, %	Luminescence, RLU	Isotope Effect *, I_D_/I_0_
Control	0	61.1 ± 4.7	–
	5	57.4 ± 4.5	–
	7.5	60.9 ± 6.0	–
	9	55.8 ± 7.1	–
	10	52.1 ± 6.7	–
NMU, 0.005M	0	1436.8 ± 144.0	–
	5	3937.8 ± 536.1	2.7
	7.5	4373.0 ± 256.7	3.0
	9	4313.8 ± 519.1	3.0
	10	2552.3 ± 759.4	1.8
MMS, 0.005M	0	320.3 ± 42.6	–
	5	547.9 ± 76.9	1.7
	7.5	1625.3 ± 190.0	5.1
	9	1759.1 ± 195.2	5.6
	10	421.0 ± 38.3	1.3

* I_D_ is the luminescence level of the culture pre-deuterated with different concentrations of D_2_O in the medium (5%, 7.5%, 9%, 10%). I_0_ is the luminescence level of non-deuterated culture.

**Table 5:** Prooxidant and antioxidant activity of the test substances on pKatG-lux and pSoxS-lux biosensors under peroxide- or paraquat-induced oxidative stress.

No.	Substance	Concen-Tration, mmol/L	Protective Activity on Biosensors, %			
			pKat-lux		pSox-lux	
			Prooxidant	Antioxidant	Prooxidant	Antioxidant
Standard antioxidants						
1	Acetylcysteine	1–30	–	94 (10) *	−11 (1)	96 (20)
2	Reduced glutathione	1–30	–	95 (10)	–	98 (30)
3	Dihydroquercetin	0.1–30	–	54 (10)	−6 (5)	46 (30)
4	Lipoic acid	0.1–6	–	72 (3)	–	76 (6)
5	Spermine	0.5–20	–	92 (2.5)	–	100 (20)
6	Lycopene	0.025–1	–	91 (1)	–	83 (0.5)
Radioprotectors						
7	Glutathione disulfide, lithium salt	0.5–10	−21 (0.5)	96 (5)	–	94 (5)
8	Glutathione disulfide magnesium salt	0.1–5	−91 (5)	–	–	7 (0.1)
9	Reduced glutathione, zinc salt	0.5–10	−81 (5)	–	–	35 (5)
10	Glutoxim	0.1–10	8 (0.5)	15 (10)	–	16 (0.5)
11	Genistein	0.5–10	8 (0.5)	45 (5)	−46 (10)	46 (0.5)
12	Androstenediol (AED)	0.001–0.1	–	27 (0.1)	−19 (0.1)	–
13	B-190 (indralin)	0.5–20	−48 (0.5)	98 (20)	–	100 (10)
14	Molixan	0.5–10	−60 (1)	–	–	29 (1)
15	Cystamine recrystallized	0.5–10	11 (0.5)	45 (10)	−37 (2.5)	–
16	Cystamine dihydrochloride	0.5–10	–	49 (10)	−28 (0.5)	–
Vitamins, amino acids						
17	Vitamin A	0.1–70	−62 (30)	–	−25 (2.5)	27 (70)
18	Vitamin E	0.1–20	–	32 (20)	−19 (0.1)	44 (20)
19	Vitamin B6	0.1–30	−12 (1)	94 (30)	−33 (5)	79 (30)
20	Vitamin PP	0.1–8	–	30 (5)	−25 (7)	–
21	Biotin	0.1–30	8 (2.5)	13 (30)	−47 (10)	–
22	Vitamin C	0.1–30	–	96 (10)	–	92 (20)
23	Cysteine	1–30	−24 (1)	96 (15)	−40 (5)	91 (20)
24	Methionine	0.1–50	−9 (15)	–	−8 (1)	30 (30)
25	Cystine	1–30	−19 (30)	–	−43 (1)	–
26	Taurine (taufon)	0.1–30	−14 (30)	16 (1)	−28 (5)	–
Substances for different purposes						
27	Mexidol	0.1–36	–	97 (36)	−33 (5)	96 (36)
28	Q10 (coenzyme)	0.1–10	9 (0.1)	18 (10)	–	69 (10)
29	DMSO	0.5–100	6 (0.5)	77 (100)	−28 (0.5)	86 (100)

* The concentrations of the substances (mmol/L) at which the specified values of prooxidant or antioxidant activity were observed are indicated in parentheses.

## Data Availability

The data is contained within the article.
